# Molecular Characterization of Thyroid Follicular Lesions in the Era of “Next-Generation” Techniques

**DOI:** 10.3389/fendo.2022.834456

**Published:** 2022-05-12

**Authors:** Esther Diana Rossi, Pietro Locantore, Carmine Bruno, Marco Dell’Aquila, Pietro Tralongo, Mariangela Curatolo, Luca Revelli, Marco Raffaelli, Luigi Maria Larocca, Liron Pantanowitz, Alfredo Pontecorvi

**Affiliations:** ^1^ Division of Anatomic Pathology and Histology, Fondazione Policlinico Universitario “Agostino Gemelli” - IRCCS, Rome, Italy; ^2^ Division of Endocrinology, Fondazione Policlinico Universitario “Agostino Gemelli” - IRCCS, Rome, Italy; ^3^ Division of Endocrine Surgery, Fondazione Policlinico Universitario “Agostino Gemelli”- IRCCS, Rome, Italy; ^4^ Department of Pathology, University of Michigan, Ann Arbor, MI, United States

**Keywords:** thyroid lesions, indeterminate categories, next-generation techniques, genetic alterations, thyroid cancers

## Abstract

It is unequivocally recognized that thyroid nodules are frequently detected in the adult population and mostly characterized by benign lesions (up to 70% of them), with only 5%–15% malignant lesions. The evaluation of thyroid lesions with fine-needle aspiration cytology (FNAC) represents one of the first and most useful diagnostic tools in the definition of their nature. Despite the fact that the majority of thyroid lesions are correctly diagnosed as either benign (70%–75%) or malignant (5%–10%) entities, the remaining nodules (20%–25%) represent the “gray zone” of follicular lesions, which belong to indeterminate categories, according to the different classification systems. This indeterminate group of lesions includes both benign and malignant entities, which cannot be easily discriminate with morphology alone. In these last decades, the increasing role of molecular testings, feasibly performed on cytological material combined with the discoveries of specific genetic alterations in the field of thyroid pathology, has opened the pace to their more accurate and specific contribution on cytology. In fact, in 2015, in the revised management guidelines for patients with thyroid nodules and well-differentiated thyroid cancers (WDTCs), the American Thyroid Association (ATA) confirmed the performance of molecular testing in thyroid indeterminate cytology, and the same performance was addressed in recent update of the management of thyroid nodules in the second edition of the Bethesda system for reporting thyroid cytopathology (TBSRTC). In the current review, we discuss the role of molecular tests for the different thyroid diagnostic categories of the Bethesda system for reporting thyroid cytopathology, mostly focusing our attention on the follicular and indeterminate lesions.

## Introduction

Fine-needle aspiration cytology (FNAC) represents the first and most important diagnostic tool for the evaluation of thyroid lesions based on its clear advantages defined by its simplicity, safety, and cost-effectiveness.

According to the literature, the majority of papers reported that more than 70% of thyroid nodules are benign, with only 5%–10% diagnosed as malignant lesions (1, 2). The cytological identification of these categories is easy enough to be confident in the correct diagnosis with the support of only morphological parameters. Nevertheless, the remaining 20%–25% of them is defined as the so-called “gray zone of indeterminate proliferations” for which a morphological discrimination is not always possible based on the fact that either benign or malignant entities are included in this indeterminate group (3–5). In these last decades, the knowledge that specific molecular mechanisms and genetics are linked with thyroid tumorigenesis and cancer has increased the performance of molecular testing on thyroid lesions on either cytology or histology. It stands to reason that molecular alterations can be translated and carried out onto clinical practice as an adjuvant tool for diagnosis, therapy, and prognosis (6, 7).

For this reason, the correct diagnosis of an indeterminate lesion is likely to benefit from the use of molecular markers for both diagnostic purposes in the discrimination of the benign and malignant nature and prognostic purposes when they may suggest a more personalized and tailored management approach.

In 2010, the first edition of the Bethesda system for reporting thyroid cytopathology (TBSRTC) represented a crucial point for a widespread international acceptance of a standardized system to classify and manage thyroid nodules by increasing the quality and reproducibility of thyroid cytology reporting (8). Although the first edition of TBSRTC did not include any mention to the possibility to test for oncogene mutations on cytological samples, the extensive literature produced about genetic alterations performed on thyroid cytology led to a revision of TBSRTC. In fact, in 2017, a second edition of TBSRTC was released addressing new topics dealing with changes in the cytomorphological criteria for FNA classification, reporting terminology (9), implied risk of malignancy (ROM) for each diagnostic category, the role of molecular testing in the different diagnostic categories, and the anticipated changes due to the recently described non-invasive follicular thyroid neoplasm with papillary-like nuclear features (NIFTP) (10).

Herein, we summarize the role of molecular application in the different categories of the II edition of TBSRTC.

### The Impact of Thyroid Diagnoses on Cytology

The molecular genetics of thyroid carcinoma can be translated into clinical practice as an ancillary tool for diagnosis and prognostication. The two most common types of thyroid carcinoma, papillary and follicular carcinoma, harbor four non-overlapping genetic alterations—*BRAF* and *RAS* point mutations and *RET/PTC* and *PAX8/PPARγ* rearrangements in more than 70% of cases (11). According to epidemiology, in the USA, thyroid cancer (TC) accounts for 6% of women cancers and less than 3% of men cancers (12). The majority are represented by well-differentiated TC (WDTC) with papillary TC (PTC) and follicular TC being at around 90% of all of TC. An early diagnosis of TC on FNAC is extremely useful mostly due to the evidence that TC has generally a very good prognosis, and 10%–15% of them are associated with recurrences and metastases including about 5% of patients with cancers not responsive to radioactive-iodine and eventually will die for the disease.

Although FNAC is able to discriminate between benign and malignant lesions in more than 80% of cases, for the remaining 20% of them, morphology alone is not able to make a conclusive diagnosis so that the application of ancillary techniques (including immunocytochemistry and molecular testing) might help in improving the performance of FNAC diagnoses and allow achieving the most appropriate surgical procedure (13).

A significant contribution to the definition of the molecular profile was offered in 2014 by the Thyroid Cancer Genome Atlas assessing that PTCs are either a *BRAF V600E* or a *RAS*-driven tumor and that these mutations are able to activate the mitogenic-activated protein kinase (MAPK) pathway (14).

In 2015, the significant contributions in the molecular field applied to thyroid pathology led to the decision by the American Thyroid Association (ATA) to publish the revised management guidelines for patients with thyroid nodules and WDTC, recommending molecular diagnostic in the cases of patients with thyroid nodules and differentiated TC with indeterminate cytology (15). The guidelines confirmed that the more significant role in performing molecular panels (including also *BRAF*, *RAS*, *RET/PTC*, and *PAX8-PPARγ*) is offered in the preoperative FNAC diagnosis in patients with indeterminate thyroid samples, supporting a possible stratification of ROM and a consistent reduction in the number of unnecessary surgical procedures (diagnostic lobectomies and/or thyroidectomies). The same suggestion was followed by the recent second TBSRTC underlining the potential role of different tests in different categories and diagnostic scenarios (16). To note, both ATA and TBSRTC did not endorse a specific and unique molecular test but suggested different molecular options in the diagnostic categories.

The role of single and specific somatic mutations, gene rearrangements, and/or microRNA (miRNA) expression profiles has been reported as having high specificity and predictive value for malignant thyroid disease (17–19). Nevertheless, in the last years, the validation of large panels rather than single mutations has shown an increasing diagnostic and prognostic role in thyroid pathology.

For example, Nikiforov et al. confirmed the advantage of using a broad next-generation sequencing (NGS) panel that provides a highly accurate, informative, and more comprehensive analysis of somatic mutations and chromosomal rearrangements in the diagnosis of nodules with AUS/FLUS and FN/SFN cytology, which ultimately facilitates the optimal management of these patients (20).

The lack of a current unique molecular approach for the cytological evaluation of thyroid nodules is balanced by two different options including the possibility of 1) in-house specific genetic panels and 2) some commercially available molecular thyroid tests. In the USA, the latter includes a) ThyroSeq (University of Pittsburgh Medical Center/Cytopath Biopsy Lab, Pittsburgh, PA), b) Afirma Gene Expression Classifier (GEC, Veracyte, South San Francisco, CA), and c) ThyGenX and ThyraMIR (both from Interpace Diagnostics, Parsippany, NJ) (21–23).

Furthermore, they have different roles. ThyroSeq and ThyGenX tests have high positive and negative predictive value (PPV and NPV) and have a role as “rule-in malignancy” based on their high PPV, whereas the Afirma GEC and the newer version gene sequencing classifier (GSC) with their high NPV help as a “rule-out malignancy” test mostly for indeterminate thyroid lesions (24). To note, GSC is a newer version of GEC, aiming to improve specificity and PPV of Afirma testing.

Herein, we discuss the clinical implication of the different molecular tests in the indeterminate Bethesda categories (**Table 1**).

**Table 1:** Characteristics of different thyroid molecular tests.

	ThyroSeq	Afirma GSC	ThyGenX	ThyraMIR
**Molecular test**	NGS	mRNA microarray analysis	Multiple PCR + mutations (somatic and rearrangements)	mRNA analysis
**NPV**	High	High	High (when used with ThyraMIR)	Scant data
**PPV**	High	Low	High (when used with ThyraMIR)	Scant data
**Sensitivity/specificity**	High/High	High/High	High/High	High/High
**Types of cytological material**	Fresh cytological samples and/or special collection	Fresh cytological samples and/or special collection	Fresh cytological samples and/or special collection	Fresh cytological samples and/or special collection
**Main relevance in their use**	Rule-in test	Rule-out test	Rule-in test	Rule-in and rule-out test
**Data analysis**	Centralized labs and/or local labs	Centralized labs	Local labs	Local labs
**Role in the cytologic categories**	Useful in the indeterminate categories, including oncocytic lesions	Useful in the indeterminate categories, including oncocytic lesions	Useful in the indeterminate categories, including oncocytic lesions	Useful in the indeterminate categories, including oncocytic lesions

NGS, next-generation sequencing; GEC, gene expression classifier; NPV, negative predictive value; PPV, positive predictive value; RT-PCR, reverse transcription polymerase chain reaction. High values were defined when > 70%.

## Molecular Characterization of Thyroid Lesions

Thyroid lesions are usually analyzed with fine needle aspiration, representing a valid diagnostic tool for the definition of the nature of these lesions. In the last decades, different classification systems have been published, each of them focusing of the opportunity to use a uniform and standardized system to allocate the lesions. Despite the different systems, they are all similar in the subclassification of indeterminate lesions in two or three subgroups, linked with their own ROM and therapeutic management. Among these classification systems, TBSRTC represents the worldwide used system. The second edition of TBSRTC Bethesda system (16) maintains the subclassification of indeterminate lesions into three different groups: AUS/FLUS, FN/SFN, and SFM with the introduction of molecular testings for some different diagnostic categories such as AUS/FLUS, FN, SFM, and malignant (M) with the purpose to contribute to a better definition of the risk stratification of thyroid nodules, as recommended by the ATA and by the National Comprehensive Cancer Network (15, 25). Nevertheless, although the role is useful for the AUS/FUS and FN/SFN, it is debated for the SFM and M categories, in which it can modify the extension of management when it is not univocally defined.

The last decades documented the performance of many different molecular approaches that can be easily adopted on cytological samples form thyroid lesions. To note, these approaches belong to the genotypic and phenotypic groups. The genotypic molecular diagnostic systems are typically based on mutation detection, whereas phenotypic methods, often referred to as classifiers, are based on RNA, both coding and noncoding RNA, or protein expression able to discriminate benign vs. malignant lesions. The latter classifiers are useful in identifying malignancy in the nodules without the definition of oncogenic mutations. However, neither approach is perfect, and a combination of both may empower the results as demonstrated in several papers with the identification of 67% of malignant nodules and 100% of benign nodules (15, 25).

### ThyroSeq

Since the first introduction of single mutational markers, among the others *BRAF* and small gene panels, the use of expanded genomic panels, multi-gene classifiers, and use of other molecular markers have highlighted the possibility of a better diagnostic accuracy, although most of these approaches still have not reached the highest possible accuracy in the detection of all main types of thyroid cancer. Among them, critical points are the correct diagnosis of oncocytic lesions, the identification of medullary TC and parathyroid lesions.

The recent discovery of additional driver mutations and gene fusions in different types of TC has opened to the development of sequencing assays able to detect multiple and various types of genetic alterations also on thyroid FNA. Nikiforov et al., in 2014, developed a NGS-based ThyroSeq test, version 2, including 56 thyroid-related genes analyzed for point mutations, gene fusions, and abnormal gene expression (26). In 2015, Nikiforov et al. applied the ThyroSeq v2.1 panel to a series of 465 AUS/FLUS cases. Their results documented that ThyroSeq v2.1 was able to classify 20/22 cancers correctly, showing a sensitivity of 90.9%, specificity of 92.1%, a PPV of 76.9%, and a NPV of 97.2%, with an overall accuracy of 91.8%. Their results assessed that the multi-gene NGS panel offers high sensitivity and high specificity for cancer detection in thyroid nodules with AUS/FLUS cytology, allowing a more personalized and tailored management (27).

The same authors analyzed a series of 143 FN/SFN with ThyroSeq v2. The results demonstrated sensitivity of 90%, specificity of 93%, a PPV of 83%, a NPV of 96%, and accuracy of 92%. In addition, for this category, NGS panel offers a highly accurate diagnosis allowing a more personalized management (26).

Similar results were also confirmed by different authors using the ThyroSeq versions for indeterminate categories. Shrestha et al., in their series of 261 cytohistological thyroid lesions, found that, in the AUS/FLUS category, mutational analysis had 85% sensitivity, 65% specificity, 50% PPV, and 91% NPV. In 12 SFN nodules analyzed with ThyroSeq panel, sensitivity was 100%, specificity was 57%, PPV was 63%, and NPV was 100%. Specifically, the prevalence of malignancy in the AUS/FLUS or SFN category was increased by nearly 15% to 45% and 53%, respectively (28).

Another paper by Valderrabano et al. included 190 AUS/FLUS and FN/SFN analyzed with ThyroSeq and documented 70% sensitivity, 77% specificity, 42% PPV, and 91% NPV. The performance of ThyroSeq v2 was significantly better in FN/SFN than that of AUS/FLUS nodules, increasing the ROM (2.5-fold), with PPV of 53%–65% (29). In another study by Zhang et al., ThyroSeq v2 in 148 indeterminate nodules reported 95% sensitivity, 60% specificity, 66% PPV, and 94% NPV (30).

**Figure 1:**
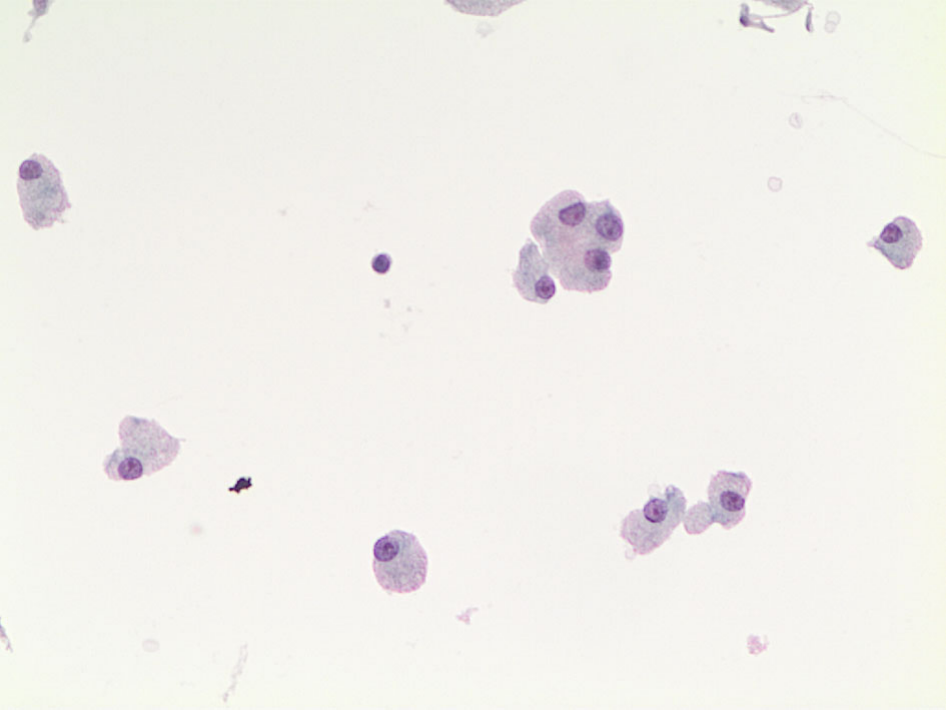
The picture shows a LBC slide of a thyroid nodule composed of oncocytic cells with the eosinophilic moderate cytoplasm, and small-to-medium nuclei with nucleolus. This thyroid nodule was diagnosed as Oncocytic Nodule (LBC 400×).

Despite the high sensitivity, specificity, and NPV of Thyroseq v2, some authors found a lower PPV. To solve this limit, Nikiforov et al. proposed a newer version, the ThyroSeq v3 with the purposes of expanding the current ThyroSeq v2 test panel by including recently discovered genetic markers related to thyroid nodules and cancer, analyzing additional classes of genetic alterations as copy number alterations (CNAs), and improving the test accuracy for detecting various types of Hurthle cell (oncocytic) tumors (**Figure 1**). The last version of ThyroSeq v.3 test, released in 2017, is a DNA- and RNA-based NGS assay, including 12,135 single-nucleotide variants and deletions/insertions in 112 genes, 120 gene fusions, 19 abnormal gene expression, and 10 CNAs in indeterminate thyroid nodules (31). Steward et al. in a study of 286 cytologic indeterminate lesions found 94% sensitivity and 82% specificity, preventing a surgical procedure in up to 61% of the patients (32). Furthermore, Desai et al. included 415 cases (AUS/FLUS, n = 251; FN/SFN, n = 164) studied with ThyroSeq v3. They found that the benign call rate (BCR) of ThyroSeq v3 for AUS/FLUS was significantly higher (82% than that for FN/SFN (54%), with also a 99.5% NPV in the AUS/FLUS category (33).

Ohori et al. evaluated the Thyroseq v3 results in a series of 224 indeterminate cases, including only AUS/FLUS and FN/SFN. They assessed that the overall BCR was 74.1% and the AUS/FLUS had a higher benign rate compared with FN/SFN. Among the mutations, *RAS* mutations were the most common positive results (34)

Chen et al. published their Canadian experience with ThyroSeq v3 in a series of 50 indeterminate lesions yielding 40% “positive” and 48% “negative” results. The additional performance of molecular testing with ThyroSeq v3 reduces the unnecessary surgery and a higher rate of malignancy (91%) in the surgical series (35).

Jun et al. compared 185 cases from ThyroSeq testing (v2 and v3) over 2 years, and they concluded that Thyroseq v3 had a lower PPV for both malignancy/NIFTP and neoplasm than v2 but did not produce any false negative results. Furthermore, even Jun et al. confirmed that *RAS-type* mutations were the most commonly identified in both benign and malignant nodules (36).

Pearlstain et al. reported a case of Hürthle cell neoplasm analyzed with ThyroSeq v3 test, showing CNAs involving multiple chromosomes with the pattern of genome haploidization more frequently found in Hürthle cell malignancy rather than Hürthle cell metaplasia or an adenoma. The histological report confirmed a 7-cm Hürthle cell carcinoma with five foci of angioinvasion found along with foci of capsular invasion, without extrathyroidal extension (37). The possibility to evaluate CNAs is important for the prediction of Hürthle cell carcinomas, which are known to have a characteristic pattern of CNAs with almost complete genome haploidization (38). In the validation study by Nikiforova et al., ThyroSeq v3 showed reliable performance in Hürthle cell cancers, with 93% sensitivity and 69% specificity (39). In a preliminary report from a recent multicenter study by David et al., ThyroSeq v3 was able to detect all HCCs (sensitivity, 100%), with all five hyperplastic nodules with Hürthle cell predominance classified as negative and overall test specificity of 66.7% (40).

**Figure 2:**
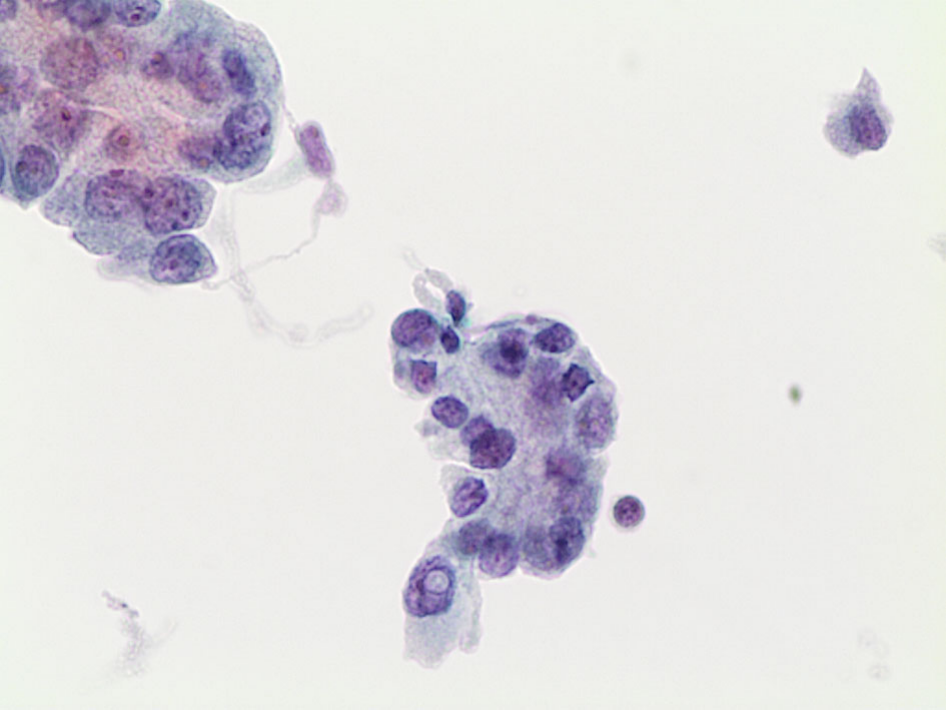
Classical morphological features of conventional PTC characterized by papillary structures and cells with atypical and pleomorphic nuclei with intranuclear pseudoinclusions (LBC 400×). This case was *BRAF V600E* mutated (LBC 400×).

In another series by Chin et al., including 50 cases with also the performance of Thyroseq v2 and/or v3, they confirmed that 64% were TC or NIFTP on surgical histopathology. Furthermore, those lesions with *TERT* or *TP53* combination mutations (*TERT/TP53*) and those with *BRAF*-like mutations were associated with a 100% probability of cancer and higher rates of extrathyroidal extension and regional nodal involvement than nodules with RAS-like mutations (**Figure 2**). These data further assessed the role of genetic panels in defining the personalized and tailored management, including extent of surgery, based on specific genetic alterations found on cytology (41).

As recently suggested by Nikiforova et al., the application of molecular testing of thyroid nodules is typically performed not only in freshly collected FNA samples but also in cytology smear slides when at least 200 to 300 cells are present. In fact, 25 smears (81%) of 31 cases were adequate for ThyroSeq analysis. The yields reported that the overall accuracy for detecting molecular alterations was 98%, with 100% concordance for mutations and gene expression alterations, 96% concordance for fusions, and 94% concordance for CNAs (42).

### ThyGenX/ThyraMIR

The Asuragen NGS technology, commercially known as miRInform (Asuragen, Austin, Tex, USA) is also known as a “newer version” of the original gene panel test to detect genetic alterations. This early version is characterized by a panel including four DNA point mutations (*BRAF*, *HRAS*, *NRAS*, and *KRAS*) and three RNA translocation fusion markers (*RET/PTC1*, *RET/PTC3*, and *PAX8/PPARγ*) with high specificity to rule-in malignancy in indeterminate thyroid nodules (23).

A more recent version of ThyGenX^®^ uses NGS to identify >100 genetic alterations across eight genes associated with thyroid malignancy. Specifically, this new version of ThyGenX is composed of the original genetic alterations/mutations analyzed in the miRInform test as well as testing for *PIK3CA* mutation, which occurs more frequently in follicular and anaplastic carcinoma.

As for its high specificity, the detection of *BRAF^V600E^* or *RET/PTC* is associated with 100% ROM, but it is lower and wider for *RAS* (range, 12%–87.5%) and *PAX8/PPARγ* (range, 50%–100%) alterations. Despite this evidence, the ROM for wild-type AUS/FLUS is only slightly higher than that of benign lesions, whereas the ROM for a wild-type FN/SFN is identical to the cases that are non-molecularly tested. To support the false negative rate of ThyGenX, the Interpace Diagnostics proposes the adjunct of ThyraMIR (from Interpace Diagnostics, Parsippany, NJ) as a reflex test, to improve the diagnostic accuracy in those cases that are not *BRAFV600E* or *RET/PTC1-3* positive (23). Different papers demonstrated that the evaluation of miRNAs has been successfully carried out on different cytological material, including indeterminate thyroid lesions, and that aberrant expression of specific miRNAs (e.g., miR-146, 221, and 222) is a clue to thyroid well-differentiated carcinomas (43–54). In fact, ThyraMIR is a thyroid microRNA (miRNA) classifier that is able to divide results into “positive” or “negative” categories.

The role of these combined testing is emphasized in two different studies enrolling indeterminate thyroid nodules (23, 55). They demonstrated high sensitivity (94% for AUS/FLUS and 82% for SFN/FN) and specificity (80% for AUS/FLUS and 91% for SFN/FN), with a PPV of 74% and a NPV of 94%. The application of multi-panel testing not only provides important information about specific mutations being present but also the prognostic relevance of some of these mutations that would suggest a total thyroidectomy also for AUS/FLUS category (23, 55, 56).

### Targeted Next-Generation Sequencing

The majority of microRNAs and molecular markers have been used and adopted to improve the preoperative risk stratification and the diagnosis of malignancy, with, on the other hand, a limited role for the definition of prognostic information in patients with confirmed malignancy. Differently from all the previous aforementioned molecular tests, targeted NGS (*RAS*, *BRAF*, *TERT*, and *P53*) or also somatic mutation-based panels add specific mutation results, implying a prognostic utility.

Different papers have emphasized that most thyroid carcinomas have an indolent course, although very few genetic alterations, such as *BRAF^V600E^* and *TERT* promoter mutations, are likely to be associated with a worse course (57). Several studies have reported a correlation between *BRAF^V600E^* and more aggressive clinicopathological outcomes of cPTC, especially with respect to bilateral disease, extrathyroidal extension, and nodal involvement (5–15). Ahmad et al. found an association of *BRAF^V600E^* mutation with extrathyroidal extension.

*TERT* promoter mutations in TC are also strongly associated with aggressive and metastatic behavior (12–21, 53). A high prevalence of *TERT* C228T mutation has been found not only in aggressive TCs, such as ATC and PDTC (24, 25, 27–29), but also in aggressive PTC variants with poor initial prognostic factors and clinical course (50).

### AFIRMA–Gene Expression Classifier and the Gene Sequencing Classifier

The Afirma GEC has been introduced to further characterize indeterminate thyroid nodules into either benign or suspicious categories. Nevertheless, data from literature have documented its relatively low PPV limited its use as a classifier for patients with suspicious results.

Afirma GEC (Veracyte, Inc., South San Francisco, CA) was developed by Veracyte, and it used a microarray measurement of messenger RNA expression of 167 genes including 142 genes in the main classifier (benign or suspicious) and 25 smaller gene expression panels to identify rare neoplasms (22).The major purpose of this test was to limit the number of unnecessary surgical procedure for benign thyroid lesions, encouraged by a high NPV similar to a benign cytological result. The data from the first study by Alexander et al. reported a NPV of 94%–95% among Bethesda III and IV nodules at a cancer prevalence of 24%–25%. The yields from different publications have assessed a range of 10%–70% fewer surgical interventions with its implementation. However, especially in patients with suspicious GEC results, the PPV was 38% and specificity was 50% (22).

Several groups studied GEC in the AUS/FLUS category confirming that those AUS/FLUS with architectural atypia have more frequently (at around 50% of cases) a negative GEC result compared with AUS/FLUS with cytologic atypia or cytologic plus architectural atypia, which are characterized by a suspicious yield. Hence, a negative GEC result reduces the ROM from 24% to 5%, supporting a simple follow-up for those patients (57). Furthermore, different papers found that patients with GEC suspicious nodules had higher ROM in cases with both architectural and nuclear atypia (57%) than in cases with architectural or nuclear atypia alone (19% and 45%, respectively) (58–61). Furthermore, an AUS/FLUS lesion with a Hürthle cell pattern endows with a low rate of GEC benign results and a very low ROM (62).

To overcome all the limitations described with GEC, the new version, a next-generation Afirma Genomic Sequencing Classifier (GSC) included not only gene expression but also the presence of DNA variants, fusions, copy number variants, and other information that may be predictive of TC (24). The new version has not altered the high original sensitivity but significantly increased its specificity limiting the number of surgical procedures to 30% of the indeterminate lesions.

As a consequence, many papers studied the comparison between GEC and GSC in the indeterminate categories.

Endo et al. compared their data from a single academic tertiary center showing an improved specificity and PPV while maintaining high sensitivity and NPV for GSC compared with GEC (63). They had an increase in benign call rates with GSC compared with GEC, and fewer false positive results, but mostly a 68% decrease of surgical procedures.

San Martin et al. compared the indeterminate categories with GEC or GSC assessing an overall surgery rate decrease from 47.8% in the GEC group to 34.7% in the GSC group (P = 0.025). They also reported a higher specificity (94% vs. 60%, P < 0.001) and PPV (85.3% vs. 40%, P < 0.001) than GEC (64).

A similar comparative evaluation was performed by Harrell et al. analyzing their data with the newer version—GSC (performed for 11 months) and also their prior experience with the GEC (performed for 86.5 months) (65). In addition, in this study, GSC reduced the number of suspicious indeterminate nodules (38.8%) as well as a decrease of oncocytic nodules classified as suspicious in the GSC group (82.7% suspicious by GEC and 35.3% classified as suspicious by GSC). As expected, significant improvements in the specificity were obtained with the oncocytic lesions and their characterization. Other results by Brauner et al. found that only 13% of GSC-suspicious nodules with HC were malignant on surgical pathology (66).

Patel et al. studied 183 indeterminate lesions with GSC resulting in a sensitivity of 91%, a specificity of 68%, a NPV of 96%, and the PPV of 47% (62). These authors concluded that GSC has a high sensitivity and accuracy for identifying benign nodules. Furthermore, the increase in the specificity offered the advantage to reduce the number of unnecessary surgery.

Angell et al. included 600 nodules with either GEC (486) or GSC (67). The benign cell rate was higher in GSC (65.8%) compared with GEC (47.9%). Furthermore, they confirmed the better performance of GSC in the field of oncocytic lesions and a higher PPV in resected nodules with a suspicious result (50% for GSC vs. 33.9% for GEC).

Geng et al. compared their experience with GEC and GSC in a series of 300 indeterminate lesions. According to their data, GSC reduces the number of unnecessary surgical procedures, as for their yields, where surgical resection could have been prevented by 61% with GSC, compared with 49% with GEC test (68).

### MicroRNA Classifiers

The basic principle of microarray platforms is defined on assessing the expression of hundreds of transcribed RNA sequences at relatively low cost. Because computational algorithms are necessary to analyze the expression patterns, these techniques may be replaced with NGS platforms. Nevertheless, miRNAs are extremely stable and remain intact in tissues and cytological material, being analyzed with feasible and reliable results in both methods. Their role has been demonstrated useful in cancer diagnosis, prognosis, and response to treatment including TC. Lithwick-Yanai et al., using the microRNA-based assay for the AUS/FLUS and FN/SFN categories, showed that the sensitivity and specificity were both 84%, with a NPV of 92% and PPV of 43% (69).

Another study by Labourier et al., performing a 10-miRNA panel and seven-gene mutational panel on a series of 109 indeterminate lesions, reported 74% PPV and 94% NPV (23).

Sistrunk et al. evaluated the clinical performance of an expanded mutation panel in combination with microRNA classification (MPTX) for the management of 140 indeterminate thyroid nodules. The MPTX results were blinded, demonstrating that a MPTX negative test status and MPTX low-risk results had a high probability (94%) of benign lesions. On the other hands, MPTX positive test with a MPTX moderate-risk results had a 53% probability of malignancy, whereas a MPTX high-risk results increased the ROM to 70% (55).

### NIFTP and Molecular Testings

The terminology of NIFTP was introduced by Nikiforov et al. in 2016 as a histological terminology defined by strict major and minor criteria. Although the histological diagnosis can be made following the criteria, the cytological identification of these NIFTP lesions has been a source of debate (10).

**Figure 3:**
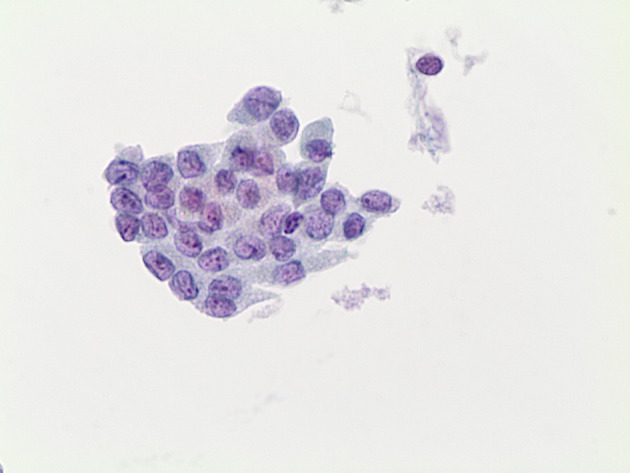
The picture shows some cluster of follicular cells characterized by pleomorphic and atypical features, including some grooves and some nuclear clarification; this case was diagnosed as suspicious for malignancy (LBC 400×) and resulted in a histological NIFTP.

For this reason, the second TBSRTC underlined that a definitive diagnosis of NIFTP cannot be possibly delivered on FNAC samples (10). Nonetheless, the detection of nuclear pseudoinclusions combined with papillary structures are typically seen in cytological samples from PTC, whereas the evidence of a predominantly follicular pattern with less frequent nuclear elongations and grooves cannot exclude a histological diagnosis of NIFTP (70). Furthermore, the possible diagnosis of NIFTP should be included among the different diagnoses in the thyroid categories. Nonetheless, different series documented that the majority of NIFTP are frequently found in the indeterminate categories with 31% in the AUS/FLUS, 26.6% in the FN/SFN and 24.3% in the SFM (**Figure 3**) (70).

The evaluation of the molecular profile in NIFTP has shown that they are similar to other follicular thyroid neoplasms. In fact, NIFTP frequently harbor *RAS* family mutations and *PAX8-PPARɤ* fusions (70–79). Nonetheless, a univocal definition of the transcriptomic landscape is not possible with the consequent difficulty to gene expression-based cytopathologic classification for NIFTP

To date, NIFTP is not linked to a specific genetic alteration (70–74). Furthermore, few recent papers assessed that NIFTP is frequently associated with suspicious Afirma GEC results (78, 79).

Although several papers found a different molecular profile between NIFTP and PTC (61, 80, 81), the same literature is not supporting the evidence that there are differences in clinic-pathological or molecular profiles between non-invasive and invasive encapsulated FVPTC cases, except with respect to vascular and capsular invasion (70–72). In their study, including 177 consecutive FVPTCs (74 non-invasive encapsulated, 51 invasive encapsulated, and 52 infiltrative), Kim et al. concluded that the molecular yields are in favor of a diagnosis of NIFPT as a neoplasm (81). In fact, any type of *RAS* mutation (*NRAS*, *HRAS*, and *KRAS* mutations) were more likely seen in encapsulated FVPTC (48.6% in non-invasive and 66.7% in invasive) than in infiltrative FVPTC (15.4%). *BRAF^V600E^* mutation confirmed to be more commonly described in the classic PTC or invasive FVPTC, as, in fact, they estimated a similar rate in non-invasive (12.2%) and invasive (11.8%) subtypes of encapsulated FVPTC and higher in infiltrative FVPTC (34.6%) (81). For other genetic alteration, i.e., *RET-PTC* rearrangements, they were exclusively found (11.5%) in infiltrative FVPTC.

According to some other authors, many NIFTP series express alterations in *RAS*, *PAX8/PPARγ*, or *BRAF^K601E^*, in contrast to the frequent *BRAF^V600E^* and *RET/PTC* alterations observed in PTC (61, 74–82). For this reason, molecular testing such as ThyroSeq v2 or ThyGenX could be a valid aid to guide an accurate cytological diagnosis and the following surgical management (total vs. hemithyroidectomy).

### Limits in the Use of Molecular Testings

Despite the performance and support of ancillary technique as a valid addition tool in the diagnosis of indeterminate lesions, it has to face some limits as the majority of molecular assays are developed on formalin-fixed paraffin-embedded (FFPE) tissue. Nevertheless, nucleic acids extracted from smears show comparable or even superior yields to those found in FFPE due to the non-formalin fixation.

The main limitations of cytological samples are related to the cytological substrates and fixatives, the limited cellularity, and the possibility to sacrifice the cytological slide without an archival slide for future review. Nevertheless, some of these limitations can be overcome by using different strategies including: 1) the use of microdissection of the tumor-rich areas, 2) the use of digital images, and 3) different cytological preparations such as cell-blocks and/or liquid-based cytology (LBC). Apart from some technical issues that can be minimized and supported by in-house laboratories adjustments, there are some cost issues. Among them, Labourier evaluated the cost-effectiveness of molecular testing in nodules with AUS/FLUS or FN/SFN cytology by using different management strategies: standard of care without molecular testing (StC), GEC, and mutation and miRNA testing (83). Their results clearly assessed that molecular testing with high benign diagnostic yield can lead to less surgeries (32% less) and cost savings (up to 67%). These results are consistent with previously reported cost–utility data and provide valuable insights for informed decision-making by patients, physicians, and payers (83).

## Conclusions

It stands to reason that, discussing the different indeterminate categories, this field of thyroid lesions is still a challenge in cytopathology practice. As emerged by the analysis of the different molecular testing, morphology is the gold standard for the diagnosis in most of these lesions, although morphology alone is not able to definitively classify all of these indeterminate lesions.

The support of ancillary molecular testing for indeterminate thyroid FNA is likely to offer some benefit in a better risk stratification of the lesions. The possibility to perform different molecular testings has been underlined by the ATA and TBSRTC because several mutation panels are both diagnostic tests and prognostic markers, and they are interchangeable in the performance on thyroid cytology. The best approach in indeterminate lesions is a combination of morphology and molecular testing to be discussed together with valuable clinical information (e.g., nodule ultrasound size and high-risk ultrasonographic characteristics) and cytomorphology.

## Author Contributions

ER, LP, and LL planned and organized the study. MD’A, MC, PL, and CB worked on the data, tables, revisions of the draft, and statistics. LR, AP, and MR contributed to the revision of draft. All authors contributed to the article and approved the submitted version.

## Conflict of Interest

The authors declare that the research was conducted in the absence of any commercial or financial relationships that could be construed as a potential conflict of interest.

## Publisher’s Note

All claims expressed in this article are solely those of the authors and do not necessarily represent those of their affiliated organizations, or those of the publisher, the editors and the reviewers. Any product that may be evaluated in this article, or claim that may be made by its manufacturer, is not guaranteed or endorsed by the publisher.
